# Associations Between Maternal Thyroid Function and Birth Outcomes in Chinese Mother-Child Dyads: A Retrospective Cohort Study

**DOI:** 10.3389/fendo.2020.611071

**Published:** 2021-02-05

**Authors:** Geng-Dong Chen, Ting-Ting Pang, Xia-Fen Lu, Peng-Sheng Li, Zi-Xing Zhou, Shao-Xin Ye, Jie Yang, Xiu-Yin Shen, Dong-Xin Lin, Da-Zhi Fan, De-Mei Lu, Zheng-Ping Liu

**Affiliations:** ^1^ Foshan Institute of Fetal Medicine, Affiliated Foshan Maternity and Child Healthcare Hospital, Southern Medical University, Foshan, China; ^2^ The Second School of Clinical Medicine, Southern Medical University, Guangzhou, China; ^3^ Department of Medical Records, Affiliated Foshan Maternity and Child Healthcare Hospital, Southern Medical University, Foshan, China; ^4^ Department of Obstetrics, Affiliated Foshan Maternity and Child Healthcare Hospital, Southern Medical University, Foshan, China

**Keywords:** free tetraiodothyronine, thyroid-stimulating hormone, thyroid peroxidase antibody, thyroid function, birth outcome

## Abstract

**Objective:**

Although research suggests a close association between maternal thyroid function and birth outcomes, no clear conclusion has been reached. We aimed to explore this potential association in a retrospective cohort study.

**Methods:**

This study included 8985 mother–child dyads. The maternal serum free tetraiodothyronine (FT4), thyroid-stimulating hormone (TSH), and thyroid peroxidase antibody (TPO Ab) concentrations and birth outcome data were reviewed from medical records. Subjects with TPO Ab concentrations of >34 and ≤34 IU/ml were classified into the TPO Ab positivity (+) and TPO Ab negativity (−) groups, respectively.

**Results:**

Compared with subjects in the normal group (0.1 ≤ TSH < 2.5 mIU/L and TPO Ab−), those with TSH concentrations of 2.5–4.0 mIU/L and TPO Ab− had a 0.65-fold lower risk of low birth weight (LBW). In contrast, those with TSH concentrations of >4.0 mIU/L, regardless of the TPO Ab status, had a 2.01-fold increased risk of LBW. Subclinical hypothyroidism, regardless of the TPO Ab status, was associated with a 1.94-fold higher risk of LBW when compared with that in subjects with euthyroidism and TPO Ab−. No other significant associations were observed.

**Conclusion:**

A maternal TSH concentration of 2.5–4.0 mIU/L was associated with a lower risk of LBW when combined with TPO Ab−, whereas subjects with a TSH concentration of >4.0 mIU/L had an increased risk of LBW. Subclinical hypothyroidism appears to be associated with a higher risk of LBW.

## Introduction

Euthyroidism, or normal thyroid function, is an important component of human health. The circulating concentrations of thyroid hormones, including free thyroxine (FT4) and thyroid-stimulating hormone (TSH), are commonly used to indicate thyroid function, whereas a concentration of anti-thyroperoxidase antibody (TPO Ab) that exceeds a certain cutoff threshold (according to measurement kits) for TPO Ab positivity (TPO Ab+) is an important factor in the diagnosis of thyroid autoimmunity. An imbalance or abnormality in these thyroid indicators can lead to overt or subclinical hyperthyroidism and hypothyroidism, which are associated with increased risks of several diseases [e.g., cardiovascular diseases (1) and overall mortality (2, 3)]. Furthermore, higher TSH concentrations are associated with an increased risk of type 2 diabetes (4), even in euthyroid subjects (5).

Adverse birth outcomes affect both the short- and long-term health of children (6, 7). Previous studies have indicated an association of maternal thyroid function with the long-term development of intelligence and brain morphology (8, 9) in offspring. Although an association of maternal thyroid function with birth outcomes has also been suggested, the results of such studies have been inconsistent, and no clear conclusions have been drawn (10–20). A recent meta-analysis of 48,145 mother–child dyads reported that maternal subclinical hypothyroidism and isolated hypothyroxinemia were respectively detrimental and protective with respect to small for gestational age (SGA) and birth weight, while higher maternal TSH and FT4 concentrations were inversely associated with birth weight. However, that study excluded TPO Ab+ subjects, and the influence of the TPO Ab status could not be determined (12). In another meta-analysis of 19 cohorts involving 47045 pregnant women, subclinical hypothyroidism, isolated hypothyroxinemia, and TPO Ab+ were associated with a high risk of preterm birth (PB). Higher risks of preterm birth were also observed in women with a TPO Ab+ status and those with TSH concentrations within the normal range or higher than 2.5 mIU/L and 4 mIU/L than in TPO Ab− women (regardless of TSH concentration) (16). However, some previous studies investigated only one or a few thyroid indicators (13, 17) or clinical status parameters (14, 18, 21), which limited a full-scale categorization and understanding of thyroid function. Additionally, thyroid functions tested at different times during gestation, and the use of different TSH cutoff points to categorize the thyroid status has increased the difficulty of comparisons across studies (10, 11, 19, 21). The 2017 Guideline of the American Thyroid Association suggested 0.1, 2.5, and 10 mIU/L as three TSH cutoff points for the diagnosis and management of thyroid disease during pregnancy (22). Recently, several studies found that TSH concentrations between 2.5 and 4.0 mIU/L or >4.0 mIU/L might exert different influences on pregnancy and birth outcomes, leading to the suggestion of a TSH concentration of 4.0 mIU/L as a potential new cutoff point for determining the thyroid function status (10, 11). Several studies also used the 2.5^th^ or 97.5^th^ percentile of TSH or FT4 (12, 16, 17), and this approach might attenuate the potential influence of race and increase the comparability of different populations. The evidence from these former studies encourages further exploration based on full-scale thyroid indicators in well-designed studies.

In this retrospective study, therefore, we aimed to investigate full-scale associations of thyroid indicators and clinical thyroid statuses (categorized using different criteria) with birth outcomes in a large population of Chinese mother–child dyads.

## Methods

**Figure 1:**
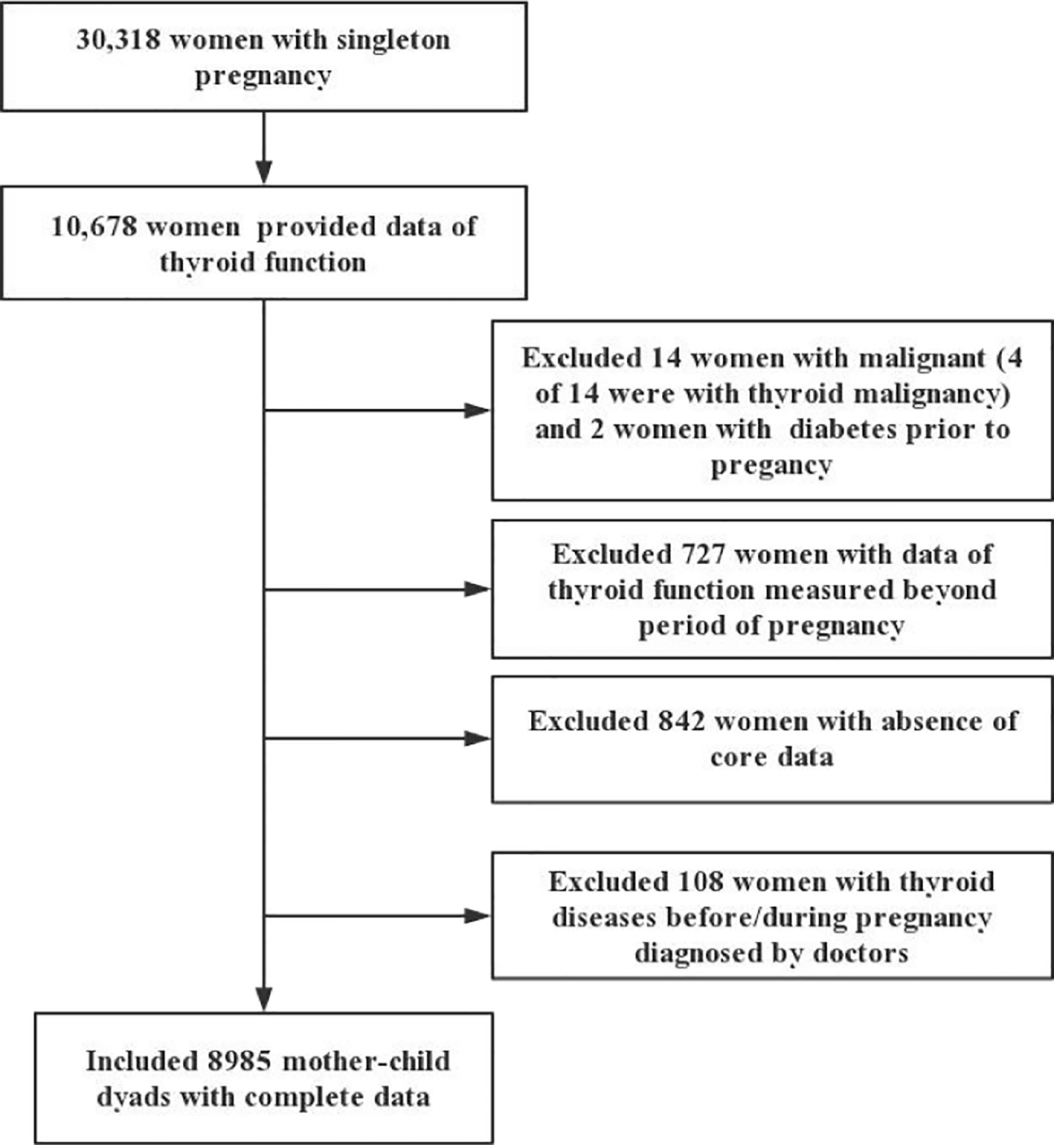
Flow chart of study participants.

This retrospective cohort study was conducted at a large obstetrics center in southern China (Foshan City, Guangdong Province). Medical records from March 1, 2015 to July 31, 2018 were reviewed. Mothers aged 18–45 years who delivered their babies at the center and underwent thyroid function testing (FT4, TSH, and TPO Ab) during pregnancy were included in this study. Subjects who met any of the following exclusion criteria were excluded: (a) twins or other multiple pregnancy; (b) history or occurrence of several serious disease, including type 2 diabetes mellitus, cardiovascular disease, and all types of cancer; (c) thyroid function measurements were only available outside of pregnancy; (d) a lack of outcome data or exposure indicators; and (e) thyroid diseases diagnosed before/during pregnancy by professional doctors through a review of the medical records. A total of 30,318 women with singleton pregnancy were available, among whom 10,678 women had available thyroid function data. After excluding 1,693 subjects due to the above-mentioned exclusion criteria (**Figure 1**), a total of 8,985 mother–child dyads with complete data were included in the study. The study protocol was approved by the ethics committee of the Affiliated Foshan Maternity and Child Healthcare Hospital, Southern Medical University. The Affiliated Foshan Maternity and Child Healthcare Hospital provided administrative permission for the research team to access and use the data included in this research.

### Thyroid Function

A universal screening of thyroid function was recommend and included as a part of regular obstetric check-ups during pregnancy in our hospital. The measurements of thyroid function were operated in our clinic chemistry and data was obtained. However, a miss of thyroid function data (indicators) would still happened if pregnant women refused to accepted these measurement (regardless of recommendation), did not followed regular obstetric check-ups, or had these measurement operated in other hospitals. Maternal blood samples were collected randomly by nurses during obstetric check-ups and analyzed quickly in the laboratory without prior freezing. Three indicators (FT4, TSH, and TPO Ab) were used to estimate maternal thyroid function during pregnancy. Serum concentrations of these indicators were measured using an electrochemical luminescence automatic immune analyzer (Cobas e601, Roche Inc., Basel, Switzerland). The gestational week when each indicator was sampled was recorded and reviewed to ensure that the maternal thyroid function was measured during pregnancy and for further use in the adjustments of statistics analysis. Subjects with TPO Ab concentrations of >34 and ≤34 IU/ml were classified into the TPO Ab+ and TPO Ab negativity (TPO Ab−) groups, respectively. Continuous indicators were divided into three groups (low: <10^th^ percentile; middle: 10^th^–90^th^ percentile; and high: >90^th^ percentile) according to their concentrations. The 10^th^ and 90^th^ cutoff values were 11.7 and 17.8 pmol/L for FT4; 0.56 and 2.75 mIU/L for TSH; and 7.80 and 32.0 IU/ml for TPO Ab, respectively, in all included subjects. The corresponding cutoff values were 11.7 and 17.8 pmol/L for FT4; 0.56 and 2.69 mIU/L for TSH; 7.63 and 23.8 IU/ml for TPO Ab, respectively, in subjects with TPO Ab−.

To accommodate both the 2017 Guidelines of the American Thyroid Association and a later suggestion that 4.0 mIU/L may be a more suitable cutoff point for determining the thyroid function status, we classified subjects into the following groups to explore the associations of different TSH concentrations in combination with the TPO Ab status: (a) TSH 0.1 to <2.5 mIU/L and TPO Ab− (reference group); (b) TSH <0.1 mIU/L and TPO Ab+/−; (c) TSH 0.1 to <2.5 mIU/L and TPO Ab+; (d) TSH 2.5–4.0 mIU/L and TPO Ab−; (e) TSH 2.5–4.0 mIU/L and TPO Ab+; (f) TSH >4.0 mIU/L and TPO Ab−; and (g) TSH >4.0 mIU/L and TPO Ab+. Only six mothers had a TSH concentration of ≥10 mIU/L and were included in groups (f) and (g). As groups (f) and (g) included only small numbers of subjects, these two groups were combined.

To enable further comparisons with the data of other studies, subjects were further divided by their clinical thyroid status using the percentile criteria used by Derakhshan et al. (12). Euthyroidism was defined as TSH and FT4 concentrations in the normal ranges (2.5^th^–97.5^th^ percentile) of our population, or 0.15–3.94 mIU/L and 10.4–21.0 pmol/L, respectively. Subclinical hypothyroidism was defined as an FT4 concentration within the normal range and a TSH concentration >97.5^th^ percentile. Overt hyperthyroidism was defined as an FT4 concentration >97.5^th^ percentile and a TSH concentration <2.5^th^ percentile, while subclinical hyperthyroidism was defined as an FT4 concentration within the normal range and a TSH concentration <2.5^th^ percentile. Only eight subjects had overt hyperthyroidism and were combined with the subclinical hyperthyroidism group. Isolated hypothyroxinemia was defined as a TSH concentration within the normal range and an FT4 concentration <2.5th percentile. We then classified subjects into the following groups to explore the associations of different clinical thyroid statuses in combination with the TPO Ab status: (a) euthyroidism and TPO Ab− (reference group); (b) euthyroidism and TPO Ab+; (c) subclinical hypothyroidism and TPO Ab+/−; (d) subclinical hyperthyroidism and TPO Ab+/−; (e) isolated hypothyroxinemia and TPO Ab+/−.

### Birth Outcomes

Two independent staff members (G.D.C and T.T.P) were responsible for extracting and reexamining the birth outcome data from medical records. The following data were collected: cesarean section (CS), preterm birth (PB), birth weight, and neonatal hyperbilirubinemia (NHB). These outcomes or diseases were diagnosed by professional physicians using the same standardization criteria. PB was defined as a delivery between 28 and <37 gestational weeks. Low birth weight (LBW) was defined as a neonatal birth weight <2,500 grams. Small-for-gestational age (SGA) and large-for-gestational age (LGA) were defined as birth weights <10^th^ and >90^th^ sex-specific percentiles, respectively, at the corresponding gestational weeks of delivery in Chinese populations (23). Appropriate-for-gestational age (AGA) was defined as a birth weight between the 10^th^ and 90^th^ sex-specific percentiles at the corresponding weeks of delivery (23).

### Other Covariates

Other potential covariates were also extracted from the medical records, including maternal age, body mass index (BMI, at delivery), gestational age, parity, delivery time, and infant sex. As gestational diabetes mellitus (GDM) was previously identified as potentially correlated with thyroid function and birth outcomes, the incidence of this condition was also recorded and treated as a potential covariate. The criteria used for the diagnosis of GDM have been reported previously (24).

### Statistical Analysis

Continuous variables are presented as medians (interquartile ranges) or means ± standard deviations (SD) according to the normality of distribution. Non-parametric tests and Student’s t-test were used to explore the differences in continuous variables between the TPO Ab+ and TPO Ab− groups. Categorical variables are presented as numbers (percentages), and differences between groups were evaluated using the chi-square test. Subjects were also divided into three groups (<10^th^, 10^th^–90^th^, and >90^th^ percentile) according to the serum thyroid hormone concentrations and stratified according to the TSH or clinical thyroid status in combination or not with the TPO Ab status, as described above.

Logistic regression analyses were used to explore the associations of the maternal thyroid function with multiple birth outcomes (CS, PB, LBW, SGA, LGA, and NHB). Two models were applied: Model 1, the univariate model, and Model 2, which was adjusted for maternal age, BMI, parity, gestational weeks, measurement time, infant sex, and GDM incidence. Adjustments for gestational weeks were excluded from the analyses of PB. AGA was used as the reference group when SGA or LGA was used as the outcome. Subjects with TPO Ab+ were excluded from the sensitivity analyses of associations between isolated thyroid indicators and birth outcomes to eliminate the potential influence of TPO Ab. All the analyses were performed using SPSS 20.0 software (Chicago, IL, USA), and a two-sided *p* value of <0.05 was considered to indicate statistical significance. The Bonferroni correction of significance was applied to multivariate logistic regression analyses if needed.

## Result

As shown in **Table 1**, 8985 mother–child dyads were included in this retrospective study. The mothers had a mean age of 30.0 ± 4.98 years, and 818 (9.1%) were TPO Ab+. Compared with TPO Ab− subjects, TPO Ab+ subjects were more likely to be older (mean age: 30.7 vs. 29.9 years, *p* < 0.001) and multiparous (parity ≥ 2: 46.9% vs. 38.5%, *p* < 0.001), had FT4 tended to be measured later in pregnancy (mean: 28.6 vs. 27.1 gestational weeks, *p* = 0.001), and had higher concentrations of TSH (median: 1.61 vs. 1.43 mIU/L, *p* < 0.001) and TPO Ab (median: 83.9 vs. 13.3 IU/ml, *p* < 0.001). In contrast, no significant differences in the BMI (p = 0.108), gestational weeks (p = 0.646), birth weight (p = 0.886), neonatal sex (p = 0.076), timing of TSH/TPO Ab measurement (p = 0.259), concentration of FT4 (p = 0.575), occurrence of GDM (p = 0.970), outcomes of CS (p = 0.230), PB (p = 0.356), LBW (p = 0.450), birth weight for gestational age (p = 0.323), or NHB (p = 0.259) were observed between TPO Ab+ and TPO Ab− subjects. A comparison of the characteristics of included and excluded subjects is shown in **Supplemental Table 1**. Among subjects with available thyroid function data, the included subjects had a lower age (p < 0.001), higher gestational age (p < 0.05), and were less likely to be multiparous (p < 0.001) than the excluded subjects. Further, the included subjects were less likely to have a PB, LBW, and LGA; a higher age, BMI, gestational age, and birthweight; and were more likely to have GDM than subjects without thyroid function data (all p < 0.05). Although statistically significant differences were detected, these differences were small (e.g., age: 30.0 vs. 29.7 years; BMI: 26.3 vs. 26.2 kg/cm^2^; gestational age: 38.8 vs. 38.7 weeks).

**Table 1:** Characteristic of subjects.

	Subclass	Total (N=8985)	TPO Ab negativity (N=8167)	TPO Ab positivity (N=818)	*P*
Age, years		30.0 ± 4.98	29.9 ± 4.96	30.7 ± 5.06	<0.001
BMI, kg/cm2		26.3 ± 3.11	26.3 ± 3.10	26.4 ± 3.16	0.108
Gestational age, weeks		38.8 ± 1.81	38.8 ± 1.82	38.8 ± 1.79	0.646
Birthweight, gram		3155 ± 443	3155 ± 440	3153 ± 466	0.886
Parity, N (%)	1	5459 (60.8)	5025 (61.5)	434 (53.1)	<0.001
	≥2	3526 (39.2)	3142 (38.5)	384 (46.9)	
Neonatal gender, N (%)	Male	4688 (52.2)	4274 (51.9)	469 (54.9)	0.076
	Female	4297 (47.8)	3965 (48.1)	385 (45.1)	
Gestational diabetes mellitus	No	7967 (88.7)	7242 (88.7)	725 (88.6)	0.970
	Yes	1018 (11.3)	925 (11.3)	93 (11.4)	
Caesarean section, N (%)	No	4913 (54.7)	4482 (54.9)	431 (52.7)	0.230
	Yes	4072 (45.3)	3685 (45.1)	387 (47.3)	
Preterm birth, N (%)	No	8468 (9.42)	7757 (94.1)	811 (95.0)	0.356
	Yes	517 (5.8)	482 (5.9)	43 (5.0)	
Low birth weight, N (%)	No	8498 (94.6)	7729 (94.6)	769 (94.0)	0.450
	Yes	487 (5.4)	438 (5.4)	49 (6.0)	
Birth weight for gestational age, N (%)	Appropriate (AGA)	7427 (82.7)	6761 (82.8)	666 (81.4)	0.323
	Small (SGA)	919 (10.2)	823 (10.1)	96 (11.7)	
	Large (LGA)	639 (7.1)	583 (7.1)	56 (6.8)	
Neonatal hyperbilirubinemia, N (%)	No	8212 (91.4)	7473 (91.5)	739 (90.3)	0.259
	Yes	773 (8.6)	694 (8.5)	79 (9.7)	
Measurement time, gestational weeks	FT4	27.2 ± 11.3	27.1 ± 11.3	28.6 ± 11.3	0.001
	TSH/TPO Ab	17.8 ± 4.29	17.8 ± 4.29	17.7 ± 4.39	0.617
Thyroid indicators	FT4, pmol/L	14.3 (12.9, 16.0)	14.4 (12.9, 16.0)	14.3 (12.9, 15.8)	0.575
	TSH, mIU/L	1.44 (0.94, 2.06)	1.43 (0.94, 2.04)	1.61 (1.05, 2.36)	<0.001
	TPO Ab, IU/mL	14.1 (9.99, 20.7)	13.3 (9.67, 18.6)	83.9 (48.3, 183)	<0.001

In univariate analyses, a higher TSH concentration (>90^th^ percentile) was significantly associated with a lower risk of PB (odds ratio [OR]: 0.62, 95% confidence interval [CI]: 0.44, 0.89; p = 0.009; reference: TSH concentration 10^th^–90^th^ percentile) (**Supplemental Table 2**). However, this association was no longer significant (after Bonferroni correction of significance) after the adjustment for potential covariates (**Table 2**). Neither higher (>90^th^ percentile) nor lower (<10^th^ percentile) thyroid function indicators were found to be associated with any of the studied birth outcomes when compared with moderate concentrations of these indicators (10^th^–90^th^ percentile). Subjects with higher concentrations (>90^th^ percentile, vs. <10^th^ percentile) of thyroid function indicators did not exhibit significantly higher or lower risks of negative birth outcomes (**Table 2**). Similar results were observed when TPO Ab+ subjects were excluded (**Supplemental Table 3**).

**Table 2:** Associations between isolated thyroid indicators and the birth outcomes.

N=8985	10^th^ - 90^th^ percentile	<10^th^ percentile		>90^th^ percentile		<10^th^ percentile	>90^th^ percentile	
	Reference	OR (95%CI)	P value^c^	OR (95%CI)	P value^c^	Reference	OR (95%CI)	P value^c^
FT4, pmol/L								
Caesarean section	1.00	0.96 (0.83, 1.11)	0.577	0.98 (0.85, 1.14)	0.817	1.00	1.03 (0.84, 1.25)	0.806
Preterm birth^a^	1.00	1.12 (0.84, 1.49)	0.453	1.03 (0.76, 1.38)	0.865	1.00	0.92 (0.62, 1.36)	0.671
Low birth weight	1.00	1.01 (0.71, 1.44)	0.955	0.64 (0.43, 0.96)	0.030	1.00	0.64 (0.38, 1.06)	0.079
Small for gestational age^b^	1.00	1.06 (0.85, 1.34)	0.595	0.92 (0.73, 1.17)	0.489	1.00	0.86 (0.63, 1.18)	0.358
Large for gestational age^b^	1.00	1.00 (0.75, 1.34)	0.985	0.99 (0.74, 1.32)	0.938	1.00	0.99 (0.67, 1.45)	0.942
Neonatal hyperbilirubinemia	1.00	0.84 (0.64, 1.09)	0.178	0.98 (0.77, 1.26)	0.889	1.00	1.18 (0.84, 1.66)	0.351
TSH, mIU/L						1.00		
Caesarean section	1.00	0.96 (0.83, 1.12)	0.609	0.96 (0.83, 1.11)	0.585	1.00	1.00 (0.82, 1.22)	0.982
Preterm birth^a^	1.00	0.91 (0.67, 1.24)	0.559	0.65 (0.45, 0.93)	0.018	1.00	0.71 (0.45, 1.12)	0.140
Low birth weight	1.00	1.09 (0.76, 1.55)	0.654	0.98 (0.68, 1.43)	0.984	1.00	0.91 (0.55, 1.49)	0.697
Small for gestational age^b^	1.00	0.84 (0.65, 1.09)	0.193	1.05 (0.84, 1.31)	0.700	1.00	1.24 (0.89, 1.72)	0.198
Large for gestational age^b^	1.00	0.91 (0.67, 1.23)	0.536	0.90 (0.66, 1.23)	0.509	1.00	0.99 (0.65, 1.50)	0.955
Neonatal hyperbilirubinemia	1.00	1.13 (0.89, 1.44)	0.311	1.00 (0.77, 1.29)	1.000	1.00	0.88 (0.63, 1.23)	0.465
TPO Ab, IU/mL						1.00		
Caesarean section	1.00	1.05 (0.90, 1.21)	0.544	0.99 (0.85, 1.14)	0.867	1.00	0.94 (0.78, 1.15)	0.561
Preterm birth^a^	1.00	1.14 (0.86, 1.51)	0.364	0.85 (0.62, 1.16)	0.302	1.00	0.74 (0.50, 1.11)	0.145
Low birth weight	1.00	1.09 (0.76, 1.56)	0.638	1.09 (0.76, 1.55)	0.647	1.00	1.00 (0.62, 1.60)	0.990
Small for gestational age^b^	1.00	0.98 (0.77, 1.23)	0.838	1.21 (0.96, 1.51)	0.103	1.00	1.24 (0.91, 1.68)	0.175
Large for gestational age^b^	1.00	0.78 (0.57, 1.08)	0.136	0.87 (0.65, 1.17)	0.364	1.00	1.11 (0.73, 1.69)	0.619
Neonatal hyperbilirubinemia	1.00	0.94 (0.72, 1.21)	0.615	1.15 (0.91, 1.46)	0.239	1.00	1.23 (0.89, 1.71)	0.215

Logistic regression: adjusted for age, BMI, parity, gestational week, gestational week of measurement, neonatal gender, gestational diabetes mellitus. ^a^variable of gestational week was excluded among the covariates in the analysis of PB. ^b^subjects with birthweight appropriate for gestational age served as the reference group. ^c^original P value before Bonferroni correction of significance. Statistical significance after Bonferroni correction of significance (α/3): P<0.0167.

Several cutoff points were used to divide subjects into several status groups according to their TSH concentrations, and the associations of different combinations of TSH and TPO Ab statuses with birth outcomes were explored. In univariate analyses (**Supplemental Table 4**), subjects with a TSH concentration of 2.5–4.0 mIU/L and TPO Ab− had a lower risk of PB (OR: 0.70, 95% CI: 0.51, 0.97) and LBW (OR: 0.70, 95% CI: 0.50, 0.99) than the subjects in the reference group (TSH 0.1 to <2.5 mIU/L with TPO Ab−). The protective association with LBW retained after a further adjustment for covariates was performed, with a corresponding OR (95% CI) value of 0.65 (0.43, 0.97). However, the protective association with PB was no longer significant after further adjustment (**Table 3**). The univariate analysis also suggested a protective association between a TSH concentration of >4.0 mIU/L with TPO Ab+/− and LGA, but this association vanished after a further adjustment for covariates. In contrast, a higher risk of LBW (OR: 2.01, 95% CI: 1.05, 3.84) was observed among subjects with a TSH concentration of >4.0 mIU/L and TPO Ab+/− (**Supplemental Table 4** and **Table 3**).

**Table 3:** Associations between thyroid-stimulating hormone (TSH) statuses combined with thyroid peroxidase antibody (TPO Ab)+/− and the birth outcomes.

	N total	Caesarean section		Preterm birth^a^		Low birth weight		Small for gestational age^b^		Large for gestational age^b^		Neonatal hyperbilirubinemia	
		N case (%)	OR(95%CI)	N case (%)	OR(95%CI)	N case (%)	OR(95%CI)	N case (%) ^b^	OR(95%CI)	N case (%) ^b^	OR(95%CI)	N case (%)	OR(95%CI)
0.1≤TSH<2.5 mIU/L and TPO Ab−	6225	3142 (45.4)	1.00	421 (6.1)	1.00	382 (5.5)	1.00	691 (10.8)	1.00	497 (8.0)	1.00	593 (8.6)	1.00
TSH<0.1 mIU/L and TPO Ab+/−	144	80 (48.5)	1.10 (0.79, 1.53)	8 (4.8)	0.75 (0.36, 1.54)	12 (7.3)	1.42 (0.71, 2.82)	21 (14.0)	1.45 (0.90, 2.34)	15 (10.4)	1.31 (0.73, 2.35)	20 (12.1)	1.47 (0.91, 2.37)
0.1≤TSH<2.5 mIU/L and TPO Ab+	560	299 (47.6)	1.01 (0.85, 1.20)	36 (5.7)	0.91 (0.64, 1.30)	38 (6.1)	1.17 (0.79, 1.75)	68 (11.6)	1.14 (0.87, 1.49)	44 (7.9)	0.94 (0.67, 1.34)	56 (8.9)	1.05 (0.79, 1.40)
2.5≤TSH ≤ 4.0 mIU/L and TPO Ab −	863	408 (42.4)	0.98 (0.85, 1.13)	42 (4.4)	0.73 (0.53, 1.02)	38 (4.0)	0.65 (0.43, 0.97)^*^	99 (11.1)	0.96 (0.76, 1.20)	68 (7.9)	1.09 (0.82, 1.44)	69 (7.2)	0.85 (0.66, 1.11)
2.5≤TSH ≤ 4.0 mIU/L and TPO Ab+	121	64 (45.7)	0.95 (0.67, 1.34)	5 (3.6)	0.58 (0.24, 1.43)	5 (3.6)	0.68 (0.25, 1.80)	19 (14.7)	1.52 (0.92, 2.52)	11 (9.1)	1.12 (0.58, 2.18)	16 (11.4)	1.39 (0.82, 2.36)
TSH>4.0 mIU/L and TPO Ab+/-	30	79 (45.4)	1.20 (0.87, 1.65)	5 (2.9)	0.46 (0.19, 1.14)	12 (6.9)	2.01 (1.05, 3.84)^*^	21 (12.4)	1.03 (0.64, 1.65)	4 (2.6)	0.41 (0.15, 1.17)	19 (10.9)	1.41 (0.87, 2.30)

Logistic regression: adjusted for age, BMI, parity, gestational week, gestational week of measurement, neonatal gender, gestational diabetes mellitus. ^a^variable of gestational week was excluded among the covariates in the analysis of PB. ^b^subjects with birthweight appropriate for gestational age served as the reference group. *P < 0.05.

We finally divided subjects into clinical thyroid status groups as indicated in the Methods. Compared with the reference group (euthyroidism and TPO Ab−), subjects with subclinical hyperthyroidism and TPO Ab+/− had a higher risk of LBW in multivariate analyses (OR: 1.94, 95% CI: 1.06, 3.55), but not in univariate analyses (**Supplemental Table 5** and **Table 4**).

**Table 4:** Associations between clinical thyroid status and the birth outcomes.

	N total	Caesarean section		Preterm birth^a^		Low birth weight		Small for gestational age^b^		Large for gestational age^b^		Neonatal hyperbilirubinemia	
		N case (%)	OR(95%CI)	N case (%)	OR(95%CI)	N case (%)	OR(95%CI)	N case (%)	OR(95%CI)	N case (%)	OR(95%CI)	N case (%)	OR(95%CI)
Euthyroidism and TPO Ab−	7605	3423 (45.0)	1.00	445 (5.9)	1.00	403 (5.3)	1.00	760 (10.8)	1.00	549 (8.0)	1.00	637 (8.4)	1.00
Euthyroidism and TPO Ab+	740	349 (47.2)	1.00 (0.86, 1.18)	40 (5.4)	0.83 (0.54, 1.28)	42 (5.7)	1.12 (0.76, 1.64)	85 (12.4)	1.22 (0.95, 1.55)	53 (8.1)	0.98 (0.71, 1.34)	71 (9.6)	1.14 (0.88, 1.48)
Subclinical hypothyroidism and TPO Ab+/−	215	98 (45.6)	1.23 (0.93, 1.64)	6 (2.8)	0.73 (0.28, 1.88)	14 (6.5)	1.94 (1.06, 3.55)^*^	27 (13.0)	1.13 (0.75, 1.72)	8 (4.3)	0.71 (0.34, 1.49)	22 (10.2)	1.33 (0.85, 2.08)
Subclinical hyperthyroidism and TPO Ab +/−	212	103 (48.6)	1.15 (0.86, 1.53)	10 (4.7)	0.78 (0.36, 1.72)	13 (6.1)	1.38 (0.73, 2.59)	26 (13.2)	1.26 (0.83, 1.94)	15 (8.1)	1.07 (0.61, 1.88)	23 (10.8)	1.33 (0.86, 2.07)
Isolated hypothyroxinemia and TPO Ab +/-	213	99 (46.5)	1.04 (0.78, 1.38)	16 (7.5)	1.48 (0.77, 2.85)	15 (7.0)	1.50 (0.81, 2.77)	21 (10.6)	0.99 (0.62, 1.58)	14 (7.3)	0.91 (0.51, 1.62)	20 (9.4)	1.13 (0.70, 1.80)

Logistic regression: adjusted for age, BMI, gestational week, neonatal gender. To concise the covariates, covariates including parity, gestational week of measurement, and gestational diabetes mellitus were excluded in the regression due to the lack of significance (P>0.05). ^a^variable of gestational week was excluded among the covariates in the analysis of PB. ^b^subjects with birthweight appropriate for gestational age served as the reference group. *P < 0.05.

## Discussion

In this retrospective cohort study of 8985 Chinese mother–child dyads, we observed few correlations of thyroid indicators and clinical statuses with birth outcomes. Compared with the reference group, subjects with a TSH concentration of 2.5–4.0 mIU/L and TPO Ab− tended to have a lower risk of LBW, whereas those with a TSH concentration of >4.0 mIU/L, regardless of the TPO Ab status, tended to have a higher risk of LBW. Subclinical hypothyroidism was associated with a higher risk of LBW, regardless of the TPO Ab status.

As noted, some researchers have suggested a TSH concentration of 4.0 mIU/L as a potential new cutoff for determining the thyroid function status (10, 11). For example, the combination of a TSH concentration between 2.5 and 4.08 mIU/L and TPO Ab− was associated with higher risks of miscarriage and maternal composite outcomes in a retrospective cohort study of 3296 Chinese mothers (10). Furthermore, a TSH concentration of >4 mIU/L (vs. ≤4 mIU/L) was associated with increased risks of neonatal respiratory distress syndrome and prematurity, but not with the risks of PB and CS, in a large retrospective cohort study of 8413 US subjects (11). Several previous studies in Europe have suggested that a TSH concentration of >2.5 mIU/L contributes to several adverse birth outcomes. In a prospective study of 1170 subjects in Greece, a higher TSH concentration (cutoff points near 2.5 mIU/L) combined with TPO Ab+ increased the risk of LBW (25). Another prospective cohort of 3988 subjects in the Netherlands observed an association between a higher TSH concentration (>2.5 mIU/L) and an increased risk of LGA in male infants (21). In contrast, in our study, a TSH concentration of 2.5–4.0 mIU/L, when combined with TPO Ab−, was associated with a lower risk of LBW. We observed a tendency toward a protective (instead of detrimental) association with PB in subjects with high TSH concentrations (>90^th^ percentile or 2.75 mIU/L), although this result did not reach statistical significance after the Bonferroni correction was applied. No significant detrimental associations with any birth outcomes were observed in subjects with TSH concentrations of 2.5–4.0 mIU/L in our study. In contrast, subjects with TSH concentrations of >4.0 mIU/L had a higher risk of LBW in our study. Our results, together with former evidence, further emphasize the potential importance of 4.0 mIU/L as a new TSH cutoff point (especially in Chinese population), as suggested in a previous study (26). Furthermore, the suitable TSH cutoff points might differ between populations, and further studies are needed to clarify the potential population-specific influence of this factor. Further potential pooling analyses should cautiously approach the application of TSH categories to different populations. Given the complex influences of TSH on different diseases and targets, more research is needed to clarify our results and explore the combination effects of TSH and TPO Ab.

We also determined that subclinical hypothyroidism with TPO Ab+/− was associated with a higher risk of LBW. Several former studies suggested an inverse association between TSH and birth weight (12) or a higher risk of LBW among mothers with subclinical hypothyroidism (12, 27). We further observed a lack of significant associations between thyroid indicators or statuses and the majority of birth outcomes, which was inconsistent with the results of former studies. For example, a large prospective cohort study of 571,785 women in Finland observed higher risks of CS, PB, and LGA in subjects with hypothyroidism (14). In two previous studies, overt hypothyroidism and hyperthyroidism were both correlated with a higher risk of PB or CS, whereas these associations were not significant among subjects with subclinical hypothyroidism or isolated hypothyroxinemia (28, 29). The evidence indicates that severe thyroid disorders might have overt detrimental effects on birth outcomes. The majority of subjects with thyroid disorders in our study were classified as subclinical, which might have reduced our ability to discover significant results.

Few previous studies explored the association between maternal thyroid function and NHB. In one study of 3507 neonates, a higher neonatal total thyroxine concentration (≥13 μg/dL) was associated with a higher risk of NHB (OR: 5.49, 95% CI: 1.19, 9.97) (30). In our study, no significant associations were observed between thyroid function and NHB. In contrast, previous studies have reported a potential link between thyroid function and NHB. Impaired thyroid function can damage liver function (31) and bilirubin metabolism (32, 33), thus increasing the risk of bilirubin accumulation. Maternal thyroid function might also contribute to the development of NHB indirectly by increasing the homocysteine concentration, which is a risk factor for jaundice (34). However, few relevant articles based on large population studies are available to elucidate the potential direct relationship between thyroid function and NHB, and no clear conclusions can be made until further studies are conducted.

This study had a few notable strengths. First, serum thyroid indicators were measured during pregnancy and before delivery, which verified the temporal sequence of events in this retrospective study. Therefore, causal inversions could be avoided. Second, multiple thyroid indicators were measured, and the large sample size enabled us to divide the population into different groups according to the TSH or clinical thyroid status in combination with the TPO Ab status. This approach provided a more comprehensive understanding of the full-scale association between thyroid function and birth outcomes. Third, the use of different TSH cutoff points, the 2.5^th^ or 97.5^th^ percentiles of thyroid indicators, and sensitivity analyses enabled the better comparison or combination of our results with those of other studies.

However, this study also had several limitations. First, it was conducted at a single obstetrics center. Although this was the largest such center in the city and covered a large population, selection bias might have been present, and further studies based on multiple obstetrics centers are needed. Second, the iodine status is known to be closely related with the thyroid function. However, the blood and urine iodine concentrations were not measured during obstetric check-ups, and this lack of data could not be compensated due to the retrospective design. Therefore, we could not eliminate the influence of the iodine status completely, although the study was performed in a southern Chinese coastal city where the residents were less likely to be iodine deficient. Third, thyroid data were measured only once, and data from multiple time points were unavailable in our study. Therefore, we were unable to explore the associations between the thyroid function status over time and birth outcomes. Fourth, data on the use of anti-thyroid drugs or thyroid hormone replacement therapy were unavailable in our study, and therefore, we could not eliminate the potential influences of these medicines. However, a total of 108 patients with thyroid diseases that had been diagnosed by doctors before/during pregnancy were excluded, as they might have been more likely to use related drugs. The remaining subjects, who did not have diagnosed thyroid diseases, might have been less likely to be exposed to related drugs. Lastly, no cases of spontaneous pregnancy loss or stillbirth were captured in our study. Therefore, we were unable to explore the associations between thyroid function and these outcomes, and our results should be interpreted carefully. Further studies are needed to address these limitations.

In conclusion, few thyroid function parameters were shown to correlate with most birth outcomes in this retrospective study. Subjects with a TSH concentration of 2.5–4.0 mIU/L and TPO Ab− tended to have lower risks of LBW, whereas those with a TSH concentration of >4.0 mIU/L had a higher risk of LBW. Subjects with subclinical hypothyroidism and TPO Ab+/− showed a detrimental association with the LBW risk. More well-designed prospective studies are needed to examine and extend these findings. Our results indicate that the maintenance of a TSH concentration of 2.5–4.0 mIU/L during pregnancy might be suitable for preventing LBW. Furthermore, the avoidance of high TSH concentrations (>4.0 mIU/L or subclinical hypothyroidism) during pregnancy might help to lower the risk of LBW. Finally, a TSH concentration of 4.0 mIU/L could be a potentially important cutoff point for evaluating thyroid function in association with birth outcomes. This topic deserves further research to explore the future clinical implications.

## Data Availability Statement

The raw data supporting the conclusions of this article will be made available by the authors, without undue reservation.

## Ethics Statement

The studies involving human participants were reviewed and approved by Ethics Committee of Affiliated Foshan Maternity & Child Healthcare Hospital, Southern Medical University. Written informed consent for participation was not required for this study in accordance with the national legislation and the institutional requirements.

## Author Contributions

G-DC, D-ML, and Z-PL devised the idea and designed the study. G-DC, X-FL, T-TP, P-SL, Z-XZ, S-XY, JY, X-YS, D-XL, and D-ZF contributed to the primary data collection. G-DC and T-TP re-examined the data and analyzed the data. G-DC and X-FL wrote the original draft, which was revised by D-ML and Z-PL. D-ML and Z-PL supervised the study and administered the project. All authors contributed to the article and approved the submitted version.

## Funding

This study was supported by the Basic and Applied Basic Research Foundation of Guangdong Province [grant numbers 2019A1515110163, G-DC], the Foundation of Bureau of Science and Technology of Foshan City [grant numbers 1920001000294, G-DC], the Foundation of Bureau of Science and Technology of Foshan City [grant numbers 2017AB002971, D-ML], and the Foshan Science and Technology Innovation Project [grant numbers 2017AG100261, Z-PL]. The funding sponsors had no role in the design of the study; in the collection, analyses, or interpretation of data; in the writing of the manuscript, and in the decision to publish the results.

## Conflict of Interest

The authors declare that the research was conducted in the absence of any commercial or financial relationships that could be construed as a potential conflict of interest.
