# Supplementary material for: Associations Between Maternal Thyroid Function and Birth Outcomes in Chinese Mother-Child Dyads: A Retrospective Cohort Study
Source: Front Endocrinol (Lausanne). 2021 Feb 5;11:611071. doi: 10.3389/fendo.2020.611071 (PMC7892893; doi:10.3389/fendo.2020.611071)
Supplement: Supplementary file 1 [file Table_1.docx]

**Supplemental Table 1.** Comparison of characteristic between included and excluded subjects.

| Items | Subclass | With data of thyroid function | | Without data of thyroid function  (N=19640) |
| --- | --- | --- | --- | --- |
|  |  | Included (N=8985) | Excluded  (N=1693) |  |
| Age, years |  | 30.0 ± 4.98 | 30.6 ± 4.37^***^ | 29.7 ± 5.11^***^ |
| BMI, kg/cm2 |  | 26.3 ± 3.11 | 26.3 ±3.13 | 26.2 ± 3.16^**^ |
| Gestational age, weeks |  | 38.8 ± 1.81 | 38.7 ± 1.79^*^ | 38.7 ± 2.17^***^ |
| Birthweight, gram |  | 3155 ± 443 | 3158 ± 429 | 3123 ± 500^***^ |
| Parity, N (%) | 1 | 5459 (60.8) | 832 (49.1)^***^ | 10305 (52.5)^***^ |
|  | ≥2 | 3526 (39.2) | 861 (50.9) | 9335 (47.5) |
| Neonatal gender, N (%) | Male | 4688 (52.2) | 880 (52.0) | 10454 (53.2) |
|  | Female | 4297 (47.8) | 813 (48.0) | 9186 (46.8) |
| Gestational diabetes mellitus | No | 7967 (88.7) | 1511 (89.2) | 17647 (89.9)^**^ |
|  | Yes | 1018 (11.3) | 182 (10.8) | 1993 (10.1) |
| Caesarean section, N (%) | No | 4913 (54.7) | 887 (52.4) | 10496 (53.4) |
|  | Yes | 4072 (45.3) | 806 (47.6) | 9144 (46.6) |
| Preterm birth, N (%) | No | 8468 (94.2) | 1594 (94.2) | 17982 (91.6)^***^ |
|  | Yes | 517 (5.8) | 99 (5.8) | 1658 (8.4) |
| Low birth weight, N (%) | No | 8498 (94.6) | 1605 (94.8) | 18115 (92.2)^***^ |
|  | Yes | 487 (5.4) | 88 (5.2) | 1525 (7.8) |
| Birth weight for gestational age, N (%) | Appropriate (AGA) | 7427 (82.7) | 1416 (83.6) | 16133 (82.2) ^*^ |
|  | Small (SGA) | 919 (10.2) | 151 (8.9) | 1953 (9.9) |
|  | Large (LGA) | 639 (7.1) | 126 (7.4) | 1551 (7.9) |
| Neonatal hyperbilirubinemia, N (%) | No | 8212 (91.4) | 1536 (90.7) | 17901 (91.1) |
|  | Yes | 773 (8.6) | 157 (9.3) | 1739 (8.9) |

^*^: *P*<0.05, ^**^: *P*<0.01, ^***^:*P*<0.001, compared with included subjects.

**Supplemental Table 2.** Univariate analyses of the associations between isolated thyroid indicators and the birth outcomes.

|  | 10^th^ - 90^th^ percentile | <10^th^ percentile | | >90^th^ percentile | |  | <10^th^ percentile | >90^th^ percentile | |
| --- | --- | --- | --- | --- | --- | --- | --- | --- | --- |
| N=8985 | *Reference* | *OR (95%CI)* | *P value* ^b^ | *OR (95%CI)* | *P value* ^b^ |  | *Reference* | *OR (95%CI)* | *P value* ^b^ |
| FT4, pmol/L |  |  |  |  |  |  |  |  |  |
| Caesarean section | 1.00 | 0.99 (0.86, 1.14) | 0.927 | 0.97 (0.84, 1.11) | 0.617 |  | 1.00 | 0.97 (0.84, 1.17) | 0.760 |
| Preterm birth | 1.00 | 1.13 (0.85, 1.50) | 0.410 | 1.02 (0.76, 1.38) | 0.887 |  | 1.00 | 0.91 (0.62, 1.34) | 0.617 |
| Low birth weight | 1.00 | 0.98 (0.72, 1.32) | 0.874 | 0.71 (0.50, 0.999) | 0.049 |  | 1.00 | 0.72 (0.47, 1.12) | 0.149 |
| Small for gestational age ^a^ | 1.00 | 0.95 (0.75, 1.20) | 0.683 | 1.04 (0.83, 1.30) | 0.738 |  | 1.00 | 0.92 (0.67, 1.25) | 0.578 |
| Large for gestational age ^a^ | 1.00 | 1.01 (0.77, 1.32) | 0.951 | 1.00 (0.76, 1.31) | 0.987 |  | 1.00 | 0.99 (0.69, 1.42) | 0.953 |
| Neonatal hyperbilirubinemia | 1.00 | 0.84 (0.65, 1.09) | 0.198 | 0.98 (0.76, 1.25) | 0.862 |  | 1.00 | 1.16 (0.83, 1.64) | 0.389 |
| TSH, mIU/L |  |  |  |  |  |  | 1.00 |  |  |
| Caesarean section | 1.00 | 1.01 (0.88, 1.16) | 0.915 | 0.86 (0.75, 0.99) | 0.034 |  | 1.00 | 0.85 (0.71, 1.03) | 0.094 |
| Preterm birth | 1.00 | 0.95 (0.70, 1.28) | 0.724 | **0.62 (0.44, 0.89)**^*^ | **0.009** |  | 1.00 | 0.66 (0.42, 1.02) | 0.062 |
| Low birth weight | 1.00 | 0.87 (0.63, 1.20) | 0.389 | 0.95 (0.70, 1.30) | 0.750 |  | 1.00 | 0.91 (0.59, 1.40) | 0.673 |
| Small for gestational age ^a^ | 1.00 | 0.79 (0.62, 1.02) | 0.065 | 1.12 (0.90, 1.39) | 0.318 |  | 1.00 | 1.41 (1.03, 1.94) | 0.031 |
| Large for gestational age ^a^ | 1.00 | 0.89 (0.67, 1.17) | 0.409 | 0.79 (0.59, 1.07) | 0.126 |  | 1.00 | 0.89 (0.61, 1.31) | 0.563 |
| Neonatal hyperbilirubinemia | 1.00 | 1.16 (0.92, 1.47) | 0.223 | 0.96 (0.74, 1.23) | 0.738 |  | 1.00 | 0.83 (0.60, 1.15) | 0.253 |
| TPO Ab, IU/mL |  |  |  |  |  |  | 1.00 |  |  |
| Caesarean section | 1.00 | 1.03 (0.90, 1.19) | 0.644 | 1.08 (0.94, 1.24) | 0.300 |  | 1.00 | 1.04 (0.87, 1.25) | 0.668 |
| Preterm birth | 1.00 | 1.16 (0.87, 1.53) | 0.311 | 0.87 (0.63, 1.19) | 0.371 |  | 1.00 | 0.75 (0.50, 1.11) | 0.155 |
| Low birth weight | 1.00 | 0.89 (0.65, 1.23) | 0.489 | 1.02 (0.75, 1.38) | 0.898 |  | 1.00 | 1.14 (0.75, 1.73) | 0.532 |
| Small for gestational age ^a^ | 1.00 | 0.98 (0.78, 1.24) | 0.892 | 1.14 (0.91, 1.42) | 0.262 |  | 1.00 | 1.15 (0.85, 1.56) | 0.353 |
| Large for gestational age ^a^ | 1.00 | 0.73 (0.54, 0.98) | 0.039 | 0.93 (0.71, 1.23) | 0.613 |  | 1.00 | 1.28 (0.87, 1.89) | 0.213 |
| Neonatal hyperbilirubinemia | 1.00 | 0.92 (0.71, 1.19) | 0.525 | 1.15 (0.91, 1.45) | 0.255 |  | 1.00 | 1.25 (0.90, 1.73) | 0.188 |

Logistic regression: univariate; ^a^: subjects with birthweight appropriate for gestational age served as the reference group. ^b^: original *P* value before Bonferroni correction of significance. ^*^: with statistical significance after Bonferroni correction of significance (α/3): P<0.0167.

**Supplemental Table 3.** Associations between isolated thyroid indicators and the birth outcomes in subjects with TPO Ab negativity .

|  |  | 10^th^ - 90^th^ percentile | <10^th^ percentile | | >90^th^ percentile | |  | <10^th^ percentile | >90^th^ percentile | |
| --- | --- | --- | --- | --- | --- | --- | --- | --- | --- | --- |
| N=8167 |  | *Reference* | *OR (95%CI)* | *P value* ^c^ | *OR (95%CI)* | *P value* ^c^ |  | *Reference* | *OR (95%CI)* | *P value* ^c^ |
| FT4, pmol/L | Caesarean section | 1.00 | 0.94 (0.81, 1.10) | 0.450 | 0.99 (0.85, 1.16) | 0.906 |  | 1.00 | 1.05 (0.86, 1.29) | 0.632 |
|  | Preterm birth ^a^ | 1.00 | 1.14 (0.85, 1.54) | 0.385 | 1.08 (0.80, 1.47) | 0.608 |  | 1.00 | 0.95 (0.64, 1.42) | 0.798 |
|  | Low birth weight | 1.00 | 1.13 (0.79, 1.62) | 0.505 | 0.67 (0.44, 1.03) | 0.067 |  | 1.00 | 0.60 (0.35, 1.01) | 0.054 |
|  | Small for gestational age ^b^ | 1.00 | 1.12 (0.88, 1.42) | 0.352 | 0.94 (0.73, 1.20) | 0.607 |  | 1.00 | 0.84 (0.60, 1.16) | 0.282 |
|  | Large for gestational age ^b^ | 1.00 | 0.93 (0.68, 1.26) | 0.628 | 0.98 (0.73, 1.33) | 0.917 |  | 1.00 | 1.06 (0.70, 1.60) | 0.773 |
|  | Neonatal hyperbilirubinemia | 1.00 | 0.84 (0.63, 1.10) | 0.206 | 1.00 (0.77, 1.30) | 0.987 |  | 1.00 | 1.19 (0.83, 1.71) | 0.337 |
| TSH, mIU/L | Caesarean section | 1.00 | 0.92 (0.79, 1.08) | 0.291 | 0.95 (0.82, 1.11) | 0.549 |  | 1.00 | 1.04 (0.84, 1.28) | 0.732 |
|  | Preterm birth ^a^ | 1.00 | 0.96 (0.70, 1.31) | 0.785 | 0.67 (0.46, 0.97) | 0.033 |  | 1.00 | 0.70 (0.44, 1.11) | 0.130 |
|  | Low birth weight | 1.00 | 1.09 (0.75, 1.58) | 0.667 | 0.83 (0.55, 1.25) | 0.368 |  | 1.00 | 0.76 (0.45, 1.30) | 0.320 |
|  | Small for gestational age ^b^ | 1.00 | 0.86 (0.66, 1.13) | 0.281 | 0.94 (0.74, 1.20) | 0.615 |  | 1.00 | 1.09 (0.77, 1.54) | 0.633 |
|  | Large for gestational age ^b^ | 1.00 | 0.91 (0.66, 1.24) | 0.538 | 0.96 (0.70, 1.33) | 0.812 |  | 1.00 | 1.06 (0.69, 1.63) | 0.786 |
|  | Neonatal hyperbilirubinemia | 1.00 | 1.12 (0.87, 1.44) | 0.394 | 1.00 (0.76, 1.31) | 0.998 |  | 1.00 | 0.90 (0.63, 1.28) | 0.538 |
| TPO Ab, IU/mL | Caesarean section | 1.00 | 1.05 (0.90, 1.23) | 0.530 | 1.01 (0.87, 1.18) | 0.862 |  | 1.00 | 0.97 (0.79, 1.19) | 0.734 |
|  | Preterm birth ^a^ | 1.00 | 1.14 (0.85, 1.53) | 0.398 | 0.83 (0.59, 1.17) | 0.285 |  | 1.00 | 0.73 (0.48, 1.12) | 0.150 |
|  | Low birth weight | 1.00 | 0.95 (0.64, 1.40) | 0.796 | 0.66 (0.43, 1.01) | 0.056 |  | 1.00 | 0.69 (0.40, 1.20) | 0.194 |
|  | Small for gestational age ^b^ | 1.00 | 0.94 (0.74, 1.21) | 0.636 | 0.82 (0.63, 1.06) | 0.126 |  | 1.00 | 0.87 (0.62, 1.22) | 0.405 |
|  | Large for gestational age ^b^ | 1.00 | 0.71 (0.50, 1.00) | 0.053 | 0.90 (0.66, 1.23) | 0.502 |  | 1.00 | 1.27 (0.81, 1.97) | 0.294 |
|  | Neonatal hyperbilirubinemia | 1.00 | 1.00 (0.76, 1.30) | 0.979 | 1.19 (0.93, 1.53) | 0.174 |  | 1.00 | 1.19 (0.85, 1.68) | 0.313 |

Logistic regression: adjusted for age, BMI, parity, gestational week, gestational week of measurement, neonatal gender, gestational diabetes mellitus. ^a^: variable of gestational week was excluded among the covariates in the analysis of PB. ^b^: subjects with birthweight appropriate for gestational age served as the reference group. ^c^: original *P* value before Bonferroni correction of significance. ^*^: with statistic significance after Bonferroni correction of significance (α/3): *P*<0.0167.

**Supplemental Table 4.** Univariate analyses of the associations between TSH statuses combined with TPO Ab+/− and the birth outcomes.

|  | N total | Caesarean section | |  | Preterm birth | |  | Low birth weight | |  | Small for gestational age ^a^ | |  | Large for gestational age ^a^ | |  | Neonatal hyperbilirubinemia | |
| --- | --- | --- | --- | --- | --- | --- | --- | --- | --- | --- | --- | --- | --- | --- | --- | --- | --- | --- |
|  |  | N case (%) | OR  (95%CI) |  | N case (%) | OR  (95%CI) |  | N case (%) | OR  (95%CI) |  | N case (%) | OR  (95%CI) |  | N case (%) | OR  (95%CI) |  | N case (%) | OR  (95%CI) |
| 0.1≤TSH<2.5 mIU/L and TPO Ab− | 6225 | 3142  (45.4) | 1.00 |  | 421 (6.1) | 1.00 |  | 382 (5.5) | 1.00 |  | 691  (10.8) | 1.00 |  | 497  (8.0) | 1.00 |  | 593  (8.6) | 1.00 |
| TSH<0.1 mIU/L and TPO Ab+/− | 144 | 80  (48.5) | 1.13  (0.83, 1.54) |  | 8 (4.8) | 0.79  (0.38, 1.61) |  | 12  (7.3) | 1.34  (0.74, 2.44) |  | 21  (14.0) | 1.35  (0.85, 2.16) |  | 15  (10.4) | 1.34  (0.78, 2.31) |  | 20  (12.1) | 1.47  (0.92, 2.37) |
| 0.1≤TSH<2.5 mIU/L and TPO Ab+ | 560 | 299  (47.6) | 1.09  (0.93, 1.29) |  | 36 (5.7) | 0.94  (0.66, 1.33) |  | 38 (6.1) | 1.10  (0.78, 1.55) |  | 68  (11.6) | 1.09  (0.84, 1.42) |  | 44  (7.9) | 0.98  (0.71, 1.36) |  | 56  (8.9) | 1.04  (0.78, 1.39) |
| 2.5≤TSH≤4.0 mIU/L and TPO Ab− | 863 | 408  (42.4) | 0.89  (0.77, 1.01) |  | 42 (4.4) | **0.70**  **(0.51, 0.97)*** |  | 38 (4.0) | **0.70**  **(0.50, 0.99)*** |  | 99  (11.1) | 1.03  (0.83, 1.29) |  | 68  (7.9) | 0.99  (0.76, 1.28) |  | 69  (7.2) | 0.82  (0.64, 1.07) |
| 2.5≤TSH≤4.0 mIU/L and TPO Ab+ | 121 | 64  (45.7) | 1.01  (0.72, 1.42) |  | 5 (3.6) | 0.57  (0.23, 1.40) |  | 5  (3.6) | 0.63  (0.26, 1.56) |  | 19  (14.7) | 1.43  (0.87, 2.35) |  | 11  (9.1) | 1.15  (0.62, 2.16) |  | 16 (11.4) | 1.38  (0.81, 2.33) |
| TSH>4.0 mIU/L and TPO Ab+/- | 30 | 79  (45.4) | 1.00  (0.74, 1.35) |  | 5 (2.9) | 0.46  (0.19, 1.12) |  | 12  (6.9) | 1.27  (0.70, 2.30) |  | 21  (12.4) | 1.17  (0.74, 1.86) |  | 4  (2.6) | **0.31**  **(0.11, 0.84)*** |  | 19  (10.9) | 1.31  (0.81, 2.12) |

Logistic regression: univariate. ^a^: subjects with birthweight appropriate for gestational age served as the reference group. *: *P*<0.05.

**Supplemental Table 5.** Univariate analyses of the associations between clinical thyroid status and the birth outcomes.

|  | N total | Caesarean section | |  | Preterm birth | |  | Low birth weight | |  | Small for gestational age ^a^ | |  | Large for gestational age ^a^ | |  | Neonatal hyperbilirubinemia | |
| --- | --- | --- | --- | --- | --- | --- | --- | --- | --- | --- | --- | --- | --- | --- | --- | --- | --- | --- |
|  |  | N case (%) | OR  (95%CI) |  | N case (%) | OR  (95%CI) |  | N case (%) | OR  (95%CI) |  | N case (%) | OR  (95%CI) |  | N case (%) | OR  (95%CI) |  | N case (%) | OR  (95%CI) |
| Euthyroidism and TPO Ab− | 7605 | 3423  (45.0) | 1.00 |  | 445  (5.9) | 1.00 |  | 403  (5.3) | 1.00 |  | 760  (10.8) | 1.00 |  | 549  (8.0) | 1.00 |  | 637  (8.4) | 1.00 |
| Euthyroidism and TPO Ab+ | 740 | 349  (47.2) | 1.09  (0.94, 1.27) |  | 40  (5.4) | 0.92  (0.66, 1.28) |  | 42  (5.7) | 1.08  (0.76, 1.49) |  | 85  (12.4) | 1.17  (0.92, 1.49) |  | 53  (8.1) | 1.01  (0.75, 1.36) |  | 71  (9.6) | 1.16  (0.90, 1.50) |
| Subclinical hypothyroidism and TPO Ab+/− | 215 | 98  (45.6) | 1.02  (0.78, 1.34) |  | 6  (2.8) | 0.46  (0.20, 1.05) |  | 14  (6.5) | 1.25  (0.72, 2.16) |  | 27  (13.0) | 1.24  (0.82, 1.88) |  | 8  (4.3) | 0.51  (0.25, 1.04) |  | 22  (10.2) | 1.25  (0.80, 1.95) |
| Subclinical hyperthyroidism and TPO Ab +/− | 212 | 103  (48.6) | 1.15  (0.88, 1.52) |  | 10  (4.7) | 0.80  (0.42, 1.51) |  | 13  (6.1) | 1.17  (0.66, 2.06) |  | 26  (13.2) | 1.26  (0.83, 1.92) |  | 15  (8.1) | 1.01  (0.59, 1.72) |  | 23  (10.8) | 1.33  (0.86, 2.07) |
| Isolated hypothyroxinemia and TPO Ab +/- | 213 | 99  (46.5) | 1.06  (0.81, 1.39) |  | 16  (7.5) | 1.31  (0.78, 2.20) |  | 15  (7.0) | 1.35  (0.79, 2.31) |  | 21  (10.6) | 0.98  (0.62, 1.55) |  | 14  (7.3) | 0.90  (0.52, 1.57) |  | 20  (9.4) | 1.13  (0.71, 1.81) |

Logistic regression: univariate. ^a^: subjects with birthweight appropriate for gestational age served as the reference group.
